# Interventions for frail community-dwelling older adults have no significant effect on adverse outcomes: a systematic review and meta-analysis

**DOI:** 10.1186/s12877-018-0936-7

**Published:** 2018-10-20

**Authors:** Michael Van der Elst, Birgitte Schoenmakers, Daan Duppen, Deborah Lambotte, Bram Fret, Bert Vaes, Jan De Lepeleire, D-SCOPE Consortium

**Affiliations:** 1 Department of Public Health and Primary Care, University of Leuven, Kapucijnenvoer 33 bus 7001, B-3000 Leuven, Belgium; 2 Department of Educational Sciences, Vrije Universiteit Brussel, Pleinlaan 2, B-1050 Brussels, Belgium; 3 Institute of Health and Society, Université Catholique de Louvain, Clos Chapelle-aux-champs 30, B-1200 Brussels, Belgium

**Keywords:** Frailty - Intervention - Community-dwelling - RCT - Review - Older adults

## Abstract

**Background:**

According to some studies, interventions can prevent or delay frailty, but their effect in preventing adverse outcomes in frail community-dwelling older people is unclear. The aim is to investigate the effect of an intervention on adverse outcomes in frail older adults.

**Methods:**

A systematic review and meta-analysis of Medline, Embase, the Cochrane Library, and Social Sciences Citation Index. Randomized controlled studies that aimed to treat frail community-dwelling older adults, were included. The outcomes were mortality, hospitalization, formal health costs, accidental falls, and institutionalization. Several sub-analyses were performed (duration of intervention, average age, dimension, recruitment).

**Results:**

Twenty-five articles (16 original studies) were included. Six types of interventions were found. The pooled odds ratios (OR) for mortality when allocated in the experimental group were 0.99 [95% CI: 0.79, 1.25] for case management and 0.78 [95% CI: 0.41, 1.45] for provision information intervention. For institutionalization, the pooled OR with case management was 0.92 [95% CI: 0.63, 1.32], and the pooled OR for information provision intervention was 1.53 [95% CI: 0.64, 3.65]. The pooled OR for hospitalization when allocated in the experimental group was 1.13 [95% CI: 0.95, 1.35] for case management. Further sub-analyses did not yield any significant findings.

**Conclusion:**

This systematic review and meta-analysis does not provide sufficient scientific evidence that interventions by frail older adults can be protective against the included adverse outcomes. A sub-analysis for some variables yielded no significant effects, although some findings suggested a decrease in adverse outcomes.

**Trial registration:**

Prospero registration CRD42016035429.

**Electronic supplementary material:**

The online version of this article (10.1186/s12877-018-0936-7) contains supplementary material, which is available to authorized users.

## Background

The population in the European Union is aging rapidly [1], and studies show that 30% of this population will be over age 65 by 2060 [1]. Therefore, the number of frail older adults with a high need for care and support will increase, and resource optimization is necessary [2, 3]. The literature describes two approaches to frailty [4–6]. The first, often designated as physical frailty, emphasizes frailty as a biological/medical concept, defined as “*a medical syndrome with multiple causes and contributors that is characterized by diminished strength, endurance, and reduced physiologic function that increases an individual’s vulnerability for developing increased dependency and/or death*” [7]. The second approach investigates frailty in a multidimensional way. In addition to strength or endurance, this perspective emphasizes cognitive, social, and psychological factors as defined by Gobbens et al.: “*Frailty is a dynamic state affecting an individual who experiences losses in one or more domains of human functioning (physical, psychological, social), which is caused by the influence of a range of variables and which increases the risk of adverse outcomes*” [8].

Many studies suggest that frailty is associated with adverse outcomes including mortality, institutionalization, hospitalization, and accidental falls [7, 9–12]. Some authors assume that early detection and intervention are important to prevent or delay frailty, improve quality of life, and reduce costs of care [7, 13]. Nevertheless, it is unclear if interventions in frail community-dwelling older adults can be protective against adverse frailty outcomes [14–18].

This systematic literature review and meta-analysis examines the following three questions: Which interventions are applied to protect frail community-dwelling older adults against adverse outcomes? What effect do interventions have on frail community-dwelling older adults in terms of mortality, hospitalization, formal health costs, accidental falls, and institutionalization? Finally, how do age, study duration, and the multi- versus unidimensional approaches of frailty and recruitment influence the effect of an intervention?

## Methods

A systematic review and meta-analysis was performed. Four electronic databases were consulted: Medline, Embase, The Cochrane Library (CL), and Social Sciences Citation Index (SSCI). The SSCI was consulted to assure that articles with a multidimensional approach to frailty would be found. The recommendations of the Cochrane Handbook for Systematic Reviews for Interventions 5.1.0 were used [19], and the protocol was registered (Prospero registration CRD42016035429).

### Search strategy

The search strategy used four key terms: aged, frail elderly, independent living, and randomized controlled trial (RCT). The final search strategy was developed with the help of a librarian (Additional file 1: Data S1). The search for articles was carried out for the first time in September 2015 and the second time on June 17, 2016. The references for the selected articles were screened for other potentially relevant publications.

### Inclusion and exclusion criteria

Within the scope of this study, the population in the included articles had to be 60 years or older, diagnosed as frail, and community-dwelling. Concerning the intervention and methodology, all studies had to be RCTs, frailty had to have been operationalized (regardless of the frailty operationalization), all types of intervention were allowed, there was no recruitment after hospital discharge (inpatient and outpatient), and the intervention must have been compared with care as usual. The studies needed to have one or more of the following outcomes: mortality, institutionalization, hospitalization, formal health costs, and accidental falls. Pilot studies and studies not written in English, French, German, or Dutch were excluded.

### Selection of studies

Retrieval and selection of studies were performed in a stepwise way. After duplicate records were removed, titles and abstracts were screened. Two reviewers assessed a sample of 12% (MVDE and DD). If their agreement reached 95%, the first author continued the inclusion process alone. In the next step, two researchers (MVDE and DD) independently read the full text of the selected articles for the inclusion and exclusion criteria. In cases of doubt or disagreement, a third researcher (BV) was asked to judge.

### Critical appraisal

Two independent researchers (DL and BF) assessed the quality of each article with the Cochrane risk of bias tool [19]. An evaluation was made in seven areas (sequence generation, allocation concealment, blinding participants and personnel, blinding of outcome assessment, incomplete outcome data, selective outcome reporting, and other bias). If an article met two or fewer criteria, it was defined as low quality; meeting three or four criteria was defined as medium quality; and if it met more than four criteria, it was considered a high-quality paper.

### Data extraction

The first author preformed the data-extraction by preparing an excel sheet including all the necessary data to answer the research questions like average age of a study, number of participants. Subsequently two researchers (DL and BF) controlled the accuracy of the data-extraction. Information concerning average age, percentage of male participants, type of intervention, operationalization of frailty, and method of recruitment of participants (e.g. participants could be recruited through census records, a service center or a care center, etc.) were subsequently collected and categorized (Additional file 2: Text S1). Frailty was defined unidimensionally if it included solely biological aspects (i.e., nutritional status, physical activity, mobility, strength, and energy). Frailty was defined multidimensionally if it also included variables such as cognition, mood, and social relations/social support [20].

### Statistics

The statistical analysis was performed with SPSS 23.0 (IBM Corp., Armonk, NY, USA) and Review Manager 5.3 [21]. For the outcomes of mortality, institutionalization (residential home/nursing home/long-term care facility), and hospitalization (inpatients), the odds ratio (OR) was calculated for every intervention; for the outcome of accidental falls, the incidence rate ratio (IRR) was presented; for formal health costs a percentage was calculated, the sum of all the presented formal health costs in the intervention group (IG) was divided by the sum of all the presented formal health costs in the control group (CG). Raw data were used for the variables mortality, institutionalization and hospitalization, for accidental falls the IRR scores were used reported in the articles. If data were unclear the first author was contacted If possible, a pooled meta-analysis was executed to measure the odds ratio. A random effect model was applied because one can assume no common effect size exist, the study population may differ from each other in ways that could affect the treatment effect (e.g., differences in the average age of the study population). Differences among included studies were assessed and described in terms of heterogeneity. A sub-analysis was performed for duration of intervention, a multi- versus unidimensional approach to frailty, average age, recruitment method of the participants, and studies with a moderate or high quality. A sub-analysis also was performed for studies that used the Fried criteria or the Frailty Index. Funnel plots were inspected, and studies with multiple research arms were analyzed separately.

## Results

The details of the search process are presented (Additional file 3: Figure S1). After the databases were searched, 25 articles were included for review representing 16 original studies. Duplicate data were excluded. All included papers are listed in Table 1 with study characteristics. The 16 original studies involved the following: nine with a case management intervention [2, 22–37], three with information provision interventions [38–41], one with physical intervention [42], one with psychosocial intervention [43], one with a pharmaceutical intervention [44], and one with a technological intervention [45]. Six articles approached frailty in an unidimensional way [2, 22–26, 37, 42, 44, 45], nine articles approached frailty in a multidimensional way [27, 29–36, 38–41, 43], and in one article the approach of frailty was unclear [28]. Two papers were of low quality (≤2) [39, 45]. For the interventions of case management and information provision, pooled meta-analyses were performed. For case management, sub-analyses also were performed.

**Table 1:** Descriptive information included articles (*N* = 16 original studies, 25 articles) continued

OS. Author	arms	N	Frailty	Dim	Intervention	Duration	Age	QA
1. Aggar (2012), Cameron (2013), Fairhall (2012, 2014 & 2015)		237	Fried	1	Case management	12	83.3	4
2. De Vriendt (2016)		168	BEL-profile scale	1	Case management	2,5	80.4	4
3. Dorrestein (2016)		359	Poor self-perceived general health, concerns about falls and related activity avoidance	2	Psychosocial intervention	4	78.3	6
4. Favela (2013)	4.1	89	Rockwood	2	Case management	9	76	3
	4.2	88	Rockwood	2	Case management	9	76	3
5. Hall (1992)		167	≥ 65 and admitted by the Long Term Care program to personal care at home	–	Case management	36	77.9	4
6. Kehusmaa (2010), Ollonqvist (2008)		708	Meet the criteria for entitlement to the SII Pensioners’ Care Allowance	2	Case management	8	78.4	4
7. Kim (2015)		66	Fried	1	Pharmaceutical intervention	3	80.7	5
8. Kono (2012 & 2013)		323	Being classified into the two lowest care need levels in the LTCI system: Support Levels 1 and 2 (out of 7)	2	Information provision intervention	24	79.9	2
9. Kono (2016)		360	Being classified into the two lowest care need levels in the LTCI system: Support Levels 1 and 2 (out of 7)	2	Information provision intervention	24	79.2	5
10. Metzelthin (2013, 2014 & 2015)		346	GFI	2	Case management	24	77.2	3
11. Monteserin (2010)		285	Meet 2 of following criteria:≥85y, > = 9 the Gijon Social Scale, ≥2 the Pfeiffer test, ≥2 the Charlson comorbidity index, ≥1 the Yesavage Depression Scale, ≥91 the Barthel index, ≥12 the Mini-Nutritional Assessment Short Form, polymedication, > 1 fall in the last 6 months and daily urinary incontinence in the last 6 months.	2	Information provision intervention	0	81.2	3
12. Perttila (2016)		83	Fried	1	Physical intervention	12	78.8	3
13. Upatising (2013)		32	Fried	1	Technological intervention	12	–	2
14. Van Hout (2010)		651	Self-reported score in the worst quartile of at least two of six COOP–WONCA charts	2	Case management	18	81.4	4
15. Van Leeuwen (2015), Hoogendijk (2016)	15.1	683	Identified by primary care physician as frail	2	Case management	6	80.6	3
	15.2	694	Identified by primary care physician as frail	2	Case management	12	80.4	3
	15.3	682	Identified by primary care physician as frail	2	Case management	18	80.8	3
16. Williams (1987)		117	No medical evaluation during the preceding year, significant decline in functional ability, unstable medical problem, unmet needs in the performance of ADL, taking three or more medications who had not had a medical evaluation within the past year, dissatisfied with current medical care, seeking a second opinion	1	Case management	8	76.5	6

*Dim* dimension of frailty: 1 = unidimensional physical/medical; 2 = multidimensional (social, cognitive, psychological) - = missing. Duration in months, age in years. Van Leeuwen et al. and Favela et al. are studies with several arms. *Ref*. = reference. *QA* Quality assessment, *OS* original study

### The effects of an intervention

The effects of an intervention in the original studies are listed in Table 2. Two results were significantly better in the IG in comparison with the CG. In Hall et al. [29], the intervention of case management resulted in a lower institutionalization, with an OR of 0.32 [95% confidence interval (CI): 0.12, 0.87]. Perttila et al. performed a study with a physical intervention, this resulted in a lower number of accidental falls with an IRR of 0.43 [42]. Four articles also offered an economic evaluation of the intervention, with one involving an information provision intervention showing a decrease in formal health costs in the IG of 11.84% in comparison with the CG [39].

**Table 2:** Results intervention on adverse outcomes

OS. Author	Mortality [CI]	Institutionalization [CI]	Health costs [CI]	Accidental falls[CI]	Hospitalization [CI]
01.Cameron et al.	1.28 [0.53, 3.09]	–	–	–	–
01.Fairhall et al.	–	–	–	1.12 [0.78, 1.63]	–
01.Fairhall et al.	–	0.83 [0.46, 1.53]	4.8%	–	1.47 [0.87, 2.47]
02.De Vriendt et al.	2.89 [0.12, 72.08]	–	–	–	–
03.Dorrestein et al.	1.20 [0.41, 3.49]	–	–	0.86 [0.65, 1.13]	–
04.Favela et al.	0.98 [0.13, 7.26]	–	–	–	–
04.Favela et al.	0.49 [0.04, 5.59]	–	–	–	–
05.Hall et al.	0.79 [0.36, 1.71]	0.32 [0.12, 0.87]	–	–	–
06.Kehusmaa et al.	0.85 [0.39, 1.83]	1.28 [0.79, 2.06]	30%	–	1.03 [0.77, 1.39]
07.Kim et al.	3.19 [0.13, 81.25]	–	–	–	–
08.Kono et al.	0.52 [0.24, 1.13]	1.70 [0.40, 7.23]	-11.8%	–	–
09.Kono et al.	1.35 [0.69, 2.64]	2.41 [0.61, 9.49]		–	–
10.Metzelthin et al.	1.21 [0.53, 2.76]	–	–	–	–
10.Metzelthin et al.	–	0.65 [0.20, 2.18]	29%	–	0.92 [0.55, 1.55]
11.Monteserin et al.	0.59 [0.24, 1.43]	0.59 [0.10, 3.56]	–	–	–
12.Perttila et al.	–	–	–	0.43 [0.33, 0.57]	–
13.Upatising et al.	7.48 [0.35, 157.7]	–	–	–	–
14.Van Hout et al.	0.86 [0.54, 1.37]	1.12 [0.60, 2.08]	–	–	1.23 [0.90, 1.68]
15.Van Leeuwen et al.	1.11 [0.50, 2.48]	–	–	–	–
15.Van Leeuwen et al.	0.88 [0.49, 1.56]	–	–	–	–
15.Van Leeuwen et al.	1.37 [0.75, 2.48]	–	–	–	–
16.Williams et al.	–	1.30 [0.33, 5.09]	–	–	1.11 [0.51, 2.42]

mortality, institutionalization, and hospitalization as odds ratio [Confidence Interval]; formal health costs as ratio intervention group relative to control group; accidental falls as IRR; double data are not reported. *OS* = original study. - = missing. Van Leeuwen et al. and Favela et al. are studies with several arms

For case management and information provision intervention, a pooled meta-analysis was performed (Table 3). The pooled ORs for mortality when allocated in the experimental group were 0.99 [95% CI: 0.79, 1.25] for case management and 0.78 [95% CI: 0.41, 1.45] for provision information intervention. The mortality ORs for the other interventions (pharmaceutical, 3.19 [95% CI: 0.13, 81.25]; psychosocial, 1.20 [95% CI: 0.41, 3.49]; technological, 7.48 [95% CI: 0.35, 157.76]) were greater than one.

**Table 3:** Odds ratio and meta-analysis of case management and information intervention provision

	Mortality [CI]	Institutionalization [CI]	Hospitalization [CI]
*Case management*			
Cameron et al. (2013)	1.28 [0.53, 3.09]	–	–
De Vriendt et al. (2016)	2.89 [0.12, 72.08]	–	–
Fairhall et al. (2015)	–	0.83 [0.46, 1.53]	1.47 [0.87, 2.47]
Favela et al. (2013)	0.98 [0.13, 7.26]	–	–
Favela et al. (2013)	0.49 [0.04, 5.59]	–	–
Hall et al. (1992)	0.79 [0.36, 1.71]	0.32 [0.12, 0.87]	–
Kehusmaa et al. (2014)	0.85 [0.39, 1.83]	1.28 [0.79, 2.06]	1.03 [0.77, 1.39]
Metzelthin et al. (2015)	1.21 [0.53, 2.76]	0.65 [0.20, 2.18]	0.92 [0.55, 1.55]
Van Hout et al. (2010)	0.86 [0.54, 1.37]	1.12 [0.60, 2.08]	1.23 [0.90, 1.68]
Van Leeuwen et al. (2015)	1.11 [0.50, 2.48]	–	–
Van Leeuwen et al. (2015a)	0.88 [0.49, 1.56]	–	–
Van Leeuwen et al. (2015b)	1.37 [0.75, 2.48]	–	–
Williams et al. (1987)	–	1.30 [0.33, 5.09]	1.11 [0.51, 2.42]
Total (95% CI)	0.99 [0.79, 1.25]	0.92 [0.63, 1.32]	1.13 [0.95, 1.35]
*Information provision intervention*			
Kono et al. (2013)	0.52 [0.24, 1.13]	1.70 [0.40, 7.23]	–
Kono et al. (2016)	1.35 [0.69, 2.64]	2.41 [0.61, 9.49]	–
Monteserin et al. (2010)	0.59 [0.24, 1.43]	0.59 [0.10, 3.56]	–
Total (95% CI)	0.78 [0.41, 1.45]	1.53 [0.64, 3.65]	–

Total = meta-analysis. - = missing. [CI] = confidence interval

For institutionalization, the pooled OR with case management was 0.92 [95% CI: 0.63, 1.32], and the pooled OR for information provision was 1.53 [95% CI: 0.64, 3.65]; they were not significant. The pooled OR for hospitalization when allocated in the experimental group was 1.13 [95% CI: 0.95, 1.35] for case management. The funnel plots, statistical heterogeneity and forest plots can be found in the appendix (Additional file 4: Figure S2: Funnel plot and Forest plot).

### Sub-analysis

The influence of duration of intervention, average age, multi- versus unidimensional approach to frailty, and recruitment on the effect of an intervention was explored. Various sub-analyses were performed but with no significant results (Table 4). For the variable of age in the category ≤80, the risk for an adverse outcome was lower. Several methods to operationalize frailty were allowed, and a sub-analysis was performed. When frailty was operationalized with the Fried criteria [5] or the Frailty Index [6], the OR for mortality was 1.12 [95% CI: 0.52, 2.41].

**Table 4:** Odds ratio or pooled odds ratio of the sub-analyses for a case management intervention for the outcomes of mortality, institutionalization, and hospitalization

	Mortality [CI]	Institutionalization [CI]	Hospitalization [CI]
*Duration (months)*			
≤6	1.18 [0.54, 2.56]	–	–
> 6 & ≤12	0.93 [0.62, 1.38]	1.10 [0.77, 1.58]	1.12 [0.88, 1.43]
> 12	1.00 [0.74, 1.37]	0.75 [0.47, 1.19]	1.14 [0.88, 1.49]
*Dimension*			
Unidimensional	1.37 [0.59, 3.18]	0.90 [0.52, 1.56]	1.35 [0.87, 2.08]
Multidimensional	0.99 [0.77, 1.27]	1.15 [0.80, 1.65]	1.09 [0.90, 1.33]
*Age (years)*			
≤80	0.90 [0.58, 1.40]	0.94 [0.65, 1.38]	1.01 [0.79, 1.29]
> 80	1.03 [0.78, 1.35]	0.96 [0.63, 1.48]	1.29 [0.99, 1.68]
*Recruitment*			
Primary health care center	1.03 [0.79, 1.36]	1.00 [0.58, 1.73]	1.14 [0.88, 1.49]
Health services	0.85 [0.50, 1.46]	0.96 [0.63, 1.45]	1.03 [0.77, 1.39]
Register	0.73 [0.16, 3.37]	–	–
Rehabilitation	1.28 [0.53, 3.09]	0.83 [0.46, 1.53]	1.47 [0.87, 2.47]
Combination	–	1.30 [0.33, 5.09]	1.11 [0.51, 2.42]

A sub-analysis was made for duration intervention, dimensional approach frailty, average population, and recruitment of the older adults. - = missing. [CI] = confidence interval

Two papers had a low quality (≤2) (Additional file 5: Figure S3: critical appraisal) [39, 45]. For information provision, a sub-analysis was performed, and the pooled OR for mortality increased from 0.76 to 0.94 [95% CI: 0.42, 2.11]. For institutionalization, the pooled OR decreased from 1.53 to 1.35 [95% CI: 0.34, 5.29] (Additional file 4: Figure S2: Funnel plot and Forest Plot).

## Discussion

The aim of this study was to investigate the effect of interventions to prevent adverse outcomes in frail community-dwelling older people. This systematic review and meta-analysis does not provide sufficient scientific evidence that interventions can be protective against the included adverse outcomes. A sub-analysis for some variables (duration of intervention, average age, dimension, recruitment) yielded no significant effects, although some findings suggested a decrease in adverse outcomes.

The results of this systematic review are in line with previous studies: the effect is unclear and inconsistent. In a systematic review, You et al. examined the effect of case management on mortality/survival days, and two out of seven articles reported a significant result [16]. Also, Hallberg and Kristerisson found in their systematic review that the effect of an intervention differed among studies: some found no effect on hospital admission, length of stay, or number of hospital days whereas others reported fewer hospital admissions and/or shorter lengths of stay [46]. Mayo-Wilson et al. concluded in their systematic review that home visiting is not consistently associated with a higher risk of mortality [47].

A pooled meta-analysis should lead to more significant and consistent results, yet this analysis did not. However, the literature provides evidence that a pooled meta-analysis could produce significant findings. For example, Elkan et al. reported that mortality and institutionalization are significantly lower after a home-based support intervention for frail older adults in comparison with a control group [48]. Thomas et al. concluded that a physical intervention reduces mortality in older community-dwelling adults, but the inclusion criteria did vary among the included studies in these two analyses, which may explain the differences in outcome. Elkan et al. included non-randomized studies and studies with older adults recently discharged from the hospital [48] whereas the older adults in Thomas et al. are not defined as frail [49].

Remarkably, the data in the current work show that the odds of being hospitalized are higher in the intervention group than in the control group (Table 2). Berglund et al. reported in their RCT that after the intervention, participants in the experimental group were much more aware of whom to contact with questions about care and services [50]. This effect could explain why the odds of being hospitalized were higher in the intervention group than in the control group and is a likely reason why the results for the outcome of formal health cost in the experimental group were not significantly lower than in the control group.

The studies in the current analysis showed heterogeneity for average age, duration, etc., which could explain the inconsistency in the results [51]. A sub-analysis should lead to significant and consistent results. For example, Stuck et al. concluded that a preventive program reduces mortality in a younger study population (mean age < 80 years) but not in older populations [52]. However, in this study, a sub-analysis for the variable average age ≤ 80 years (Table 3) was not significant, and neither were the results of other sub-analyses. Our findings confirm Elkan et al.: population type, duration, and age have no significant effect on mortality and institutionalization [48].

### Considerations for future research

A plausible reason for the lack of evidence is the heterogeneity within studies. Within studies, the contextual factors of the population in the experimental group was heterogeneous, with differences in age, educational level, morbidities, and context, etc. If frailty is operationalized with a multidimensional approach, however, the question that arises is: ‘which dimensions were problematic?’ Also the local setting within studies was heterogeneous, Van Leeuwen et al. used two regions, and Kono et al. and Perttila et al. used three regions [36, 39, 40, 42]. It is plausible that an intervention within a subgroup is effective. Analyzing the results of an experiment on an aggregated level might lead to an ecological fallacy [53].

Future research should not solely focus on the effect of an intervention but also address the question: why did interventions work when they did or why not, for who did they work and what contextual factors triggered the mechanisms required to make them work. This is described by Pawson and Tilley in ‘realistic evaluation’ (1997). They suggest that a realistic evaluation approach might provide a better understanding of the effect of an intervention [54]. This approach is a theory-driven method that not only addresses the outcome of an intervention, but also why interventions worked, when they worked or for who they worked [54].

A consensus about the concept of frailty is necessary for future research and would enable comparison, evaluation, and replication of interventional studies. Some authors have made valuable efforts toward reaching a consensus [7]; for example, ‘The White Book on Frailty’ has delivered an important contribution to this understanding [13].

Several other explanations are possible for the current results. The selected population may have been detected too late and already have been too frail [13, 15]. In addition, societal trends, such as changes in structure and function of families, might have aggravated the incidence or severity of frailty and complicated its effective management [13]. A lack of mindfulness for these societal trends also may be an explanation for the non-significant results. Several authors have discussed the difficulties of implementing the intervention [32, 34]. As a last consideration, future research making an economic evaluation must consider the extra awareness of services that older adults gain through an intervention [50].

### Strengths and limitations

Previous systematic reviews have focused on the effect of one intervention in comparison with care as usual [55–58]. A strength of the current analysis is the overview of interventions for frail community-dwelling older adults in the context of several adverse outcomes. A second strength is that only RCTs were included whereas several other systematic reviews have also included non-RCTs [46, 51]. In this analysis, differences among studies were assessed (heterogeneity) in terms of duration of the intervention, average participant age, dimensional approach to frailty, recruitment of participants, and frailty operationalization, constituting a third strength. A fourth strength is that three of the five outcome measures – mortality, institutionalization, and hospitalization – are collected primarily through registers and can be seen as objective data, which decreases the risk for bias [2].

The analysis also has some weaknesses, so that the results should be interpreted with caution. A first weakness is the small number of original studies, which led to meta-analyses only for case management and information provision and reduced the reliability of the results. One reason for the small numbers of included publications is the lack of operationalization of ‘frailty’ in studies. An absence of an operationalization of frailty is also a feature in other studies [15, 17]. Other reasons for exclusion were a lack of usual care, no relevant outcomes, and the recruitment of non-community-dwelling participants*.* A second weakness is the concept of frailty. Several methods are used to operationalize frailty, and some may not be accurate enough to recruit frail older adults, making study comparison and evaluation difficult [56]. A third weakness is that several concepts, such as case management, information provision, institutionalization, and formal health costs, have different operationalizations, leading to heterogeneity among studies. In the current analysis, mortality, institutionalization, accidental falls, formal health costs, and hospitalization were used because they are often cited as adverse outcomes. Other outcomes not included in this systematic review include functional status, physical performances, quality of life, mastery, disability, etc. [14], which can be seen as a weakness. These outcomes are not included because of the different methods to operationalize these concepts.

## Conclusion

The number of frail older adults with a high need of care and support is increasing. According to some studies, interventions can prevent or delay frailty, but their effect in preventing adverse outcomes in frail community-dwelling older people is unclear. The aim of this article was to investigate if interventions for frail community-dwelling older adults can be protective against adverse outcomes. This systematic review and meta-analysis does not provide sufficient scientific evidence that supports this assumption, even though some results suggest a decrease in adverse outcomes.

Future research must consider that the research population of older adults is very heterogeneous, also within studies. A good breakdown of all of these characteristics is necessary, and sub-analyses might avoid ecological fallacies. Each patient’s specific needs and how to deliver these services are probably essential for the effectiveness of an intervention. New methods/approaches, for example the realist approach might provide a better understanding of the effect of an intervention. Future research must also consider new societal trends, implementation problems, and heightened awareness about services that may influence the results.

## Additional files

Additional file 1: **Data S1.** Search strategy (DOCX 15 kb)

Additional file 2: **Text S1.** Extra information operationalization and categorization of Variables. (DOCX 19 kb)

Additional file 3: **Figure S1.** Flow chart (PDF 188 kb)

Additional file 4: **Figure S2.** Funnel plot and Forest plot (PDF 257 kb)

Additional file 5: **Figure S3.** Critical appraisal (PDF 187 kb)

## Abbreviations

CG: Control group
CI: Confidence interval
CL: The Cochrane Library
IG: Intervention group
IRR: Incidence rate ratio
OR: Odds ratio
OS: Original studies
RCT: Randomized controlled trial
SSCI: Social Sciences Citation Index
