# Supplementary material for: Interventions for frail community-dwelling older adults have no significant effect on adverse outcomes: a systematic review and meta-analysis
Source: BMC Geriatr. 2018 Oct 20;18:249. doi: 10.1186/s12877-018-0936-7 (PMC6195949; doi:10.1186/s12877-018-0936-7)
Supplement: Supplementary file 1 — Data S1. Search strategy (DOCX 15 kb) [file 12877_2018_936_MOESM1_ESM.docx]

**Addidtional Data S1: Search strategy**

**Medline**

((("Frail Elderly"[MeSH] OR (("Aged"[Mesh:noexp] OR aged[tiab] OR aging[tiab] OR ageing[tiab] AND elder*[tiab] AND "Aged, 80 and over"[MeSH] OR oldest old[tiab] OR old*[tiab] OR senior*[tiab] OR geriatric*[tiab] OR "Veterans"[MeSH] OR veteran*[tiab]) AND (frail*[tiab] OR weakness[tiab] OR weak[tiab] OR fragil*[tiab] OR vulnerab*[tiab] OR unhealth*[tiab] OR debil*[tiab] OR "functional impairment"[tiab])))) AND ("home care service"[Mesh] OR Domiciliary Care[tiab] OR home care*[tiab] OR home[tiab] OR "homecare"[tiab] OR "home based care"[tiab] OR "community dwelling"[tiab] OR "Independent Living"[MeSH] OR independent*[tiab] OR "Intermediate Care Facilities"[Mesh] OR "aging in place"[tiab] OR "congregate living facilities"[tiab] OR "congregate living facility"[tiab])) AND ((((randomized controlled trial[pt] OR controlled clinical trial[pt] OR randomized[tiab] OR placebo[tiab] OR drug therapy[sh] OR randomly[tiab] OR trial[tiab] OR groups[tiab]))) NOT (animals[mh] NOT humans[mh]))

**The Cochrane Library**

1. Frail Elderly[MeSH]
2. Aged[MeSH:noexp]
3. aged[tiab]
4. aging[tiab]
5. ageing[tiab]
6. elder*[tiab]
7. Aged, 80 and over[MeSH]
8. oldest old[tiab]
9. old*[tiab]
10. senior*[tiab]
11. geriatric*[tiab]
12. Veterans[MeSH]
13. veteran*[tiab]
14. 1 OR 2 OR 3 OR 4 OR 5 OR 6 OR 7 OR 8 OR 9 OR 10 OR 11 OR 12 OR 13
15. frail*[tiab]
16. weakness[tiab]
17. weak[tiab]
18. fragil*[tiab]
19. vulnerab*[tiab]
20. unhealth*[tiab]
21. debil*[tiab]
22. functional impairment[tiab]
23. 15 OR 16 OR 17 OR 18 OR 19 OR 20 OR 21 OR22
24. home care service[MeSH]
25. Domiciliary Care[tiab]
26. home care*[tiab] OR home[tiab]
27. homecare[tiab]
28. home based care[tiab]
29. community dwelling[tiab]
30. Independent Living[MeSH]
31. independent*[tiab]
32. Intermediate Care Facilities[MeSH]
33. aging in place[tiab]
34. congregate living facilities[tiab]
35. congregate living facility[tiab]
36. 24 OR 25 OR 26 OR 27 OR 28 OR 29 OR 30 OR 31 OR 32 OR 33 OR 34 OR 35
37. 23 AND 36
38. randomized controlled trial[pt]
39. controlled clinical trial[pt]
40. randomized[tiab]
41. placebo[tiab]
42. drug therapy[sh]
43. randomly[tiab]
44. trial[tiab]
45. groups[tiab]
46. 38 OR 39 OR 40 OR 41 OR 42 OR 43 OR 44 OR 45
47. 37 AND 46
48. animals[mh]
49. humans[mh]
50. 48 NOT 49
51. 50 AND 51

**Embase**

frail elderly'/exp OR ('aged'/de OR 'veteran'/exp OR 'very elderly'/exp OR 'aged':ab,ti OR 'aging':ab,ti OR 'ageing':ab,ti OR elder*:ab,ti OR 'oldest old':ab,ti OR old*:ab,ti OR senior*:ab,ti OR geriatric*:ab,ti OR veteran*:ab,ti AND (frail*:ab,ti OR weak:ab,ti OR weakness:ab,ti OR fragil*:ab,ti OR vulnerab*:ab,ti OR unhealth*:ab,ti OR debil*:ab,ti OR 'functional impairment':ab,ti)) AND ('home care'/exp OR 'domiciliary care':ab,ti OR home:ab,ti OR 'home care':ab,ti OR 'homecare':ab,ti OR 'home based care':ab,ti OR 'community dwelling':ab,ti OR 'independent living'/exp OR 'independent':ab,ti OR 'intermediate care facilities':ab,ti OR 'aging in place':ab,ti OR 'congregate living facilities':ab,ti OR 'congregate living facility':ab,ti) AND ('randomized controlled trial (topic)' OR 'controlled clinical trial (topic)' OR 'randomized':ab,ti OR 'placebo':ab,ti OR 'drug therapy':ab,ti OR randomly:ab,ti OR trial:ab,ti OR groups:ab,ti) NOT ('animals':ab,ti NOT 'human':ab,ti) AND 'article'/it

**SSCI**

1. ("aged" OR "aging" OR "ageing" OR "very elderly" OR elder* OR "oldest old" OR old* OR senior* OR geriatric* OR veteran*)

2. ("frail elderly" OR frail* OR "weak" OR "weakness" OR fragil* OR vulnerab* OR unhealth* OR debil* OR "functional impairment")

3. ("randomized" OR "controlled clinical trial" OR "randomly" OR "randomized controlled trial" OR "groups" OR "trial" OR "drug therapy" OR "placebo")

4. ("home care service" OR "domiciliary care" OR "home care" OR "homecare" OR "home" OR "home based care" OR "community dwelling" OR "independent living" OR "intermediate care facilities" OR "congregate living facilities" OR "aging in place")

#4 AND #3 AND #2 AND #1
