# Supplementary material for: Interventions for frail community-dwelling older adults have no significant effect on adverse outcomes: a systematic review and meta-analysis
Source: BMC Geriatr. 2018 Oct 20;18:249. doi: 10.1186/s12877-018-0936-7 (PMC6195949; doi:10.1186/s12877-018-0936-7)
Supplement: Supplementary file 2 — Text S1. Extra information operationalization and categorization of Variables. (DOCX 19 kb) [file 12877_2018_936_MOESM2_ESM.docx]

**Additional Text S1: Extra information operationalization and categorization of Variables.**

Age: The mean age of the total study population. The mean age of the total study population was calculated if the data for the experimental and control groups were given. A weighted method was used. If the data were given as a categorical variable, the mean age was calculated by a proxy. ∑(Lower age + upper age)/2*N.

Intervention

The development of the interventions started with a search in PubMed in August 2015. The search strategy was frailty*RCT*last 5 years. This resulted in 102 articles. The 102 articles were categorized into six types of interventions.

Definitions

Case management – a collaborative process of assessment, planning, facilitation, care coordination, evaluation, and advocacy for options and services to meet an individual’s and family’s comprehensive health needs through communication and available resources to promote quality, cost-effective outcomes (1).

According to Van Durme et al. (2), case management interventions include four of the six elements of the definition of the Case Management Society of America (CMSA). In this study, an intervention was categorized as case management if it included four of the six elements of the definition.

No distinction between case management and integrated care was made. There were overlaps between the different models of care. Integrated care models usually included case management.

Information provision intervention (MeSH) – information intended for potential users of medical and healthcare services. There is an emphasis on self-care and preventive approaches as well as information for community-wide dissemination and use.

Psychosocial intervention – includes the broad spectrum of treatments of complaints that are not strictly medical or somatic. On the one hand, these interventions deal with various psychological problems such as anxiousness, nervousness, tenseness (posttraumatic or acute) stress, depression and feeling depressed, burn out, loneliness, and irritability. On the other hand, these interventions concern various social problems such as poverty/financial problems, housing problems, problems with social security or health care, adjustment problems, and loss/death of family/partner (3).

Pharmaceutical intervention – an intervention that uses drugs or supplements, etc., and that prospectively assigns human participants or groups of humans to a health-related intervention with drugs/supplements to evaluate the effects on health outcomes (4).

Technological intervention – (1) an intervention with devices, not affixed to the body, designed to help persons having musculoskeletal or neuromuscular disabilities to perform activities involving movement (MeSH) ; or (2) an intervention with telemedicine, the use of electronic information and communications technologies to provide and support health care when distance separates the participants (5).

Physical intervention – any bodily movement produced by skeletal muscles that requires energy expenditure (6).

Primary health care center – first-contact, accessible, continued, comprehensive, and coordinated care. First-contact care is accessible at the time of need; ongoing care focuses on the long-term health of a person rather than on the short duration of the disease; comprehensive care is a range of services appropriate to the common problems in the respective population, and coordination is the role by which primary care acts to coordinate other specialists that the patient may need (7).

Home services – (1) home care services (MeSH): community health and nursing services providing coordinated multiple services to the patient at the patient’s home, provided by a visiting nurse, home health agencies, hospitals, or organized community groups using professional staff for care delivery ; or (2) homemaker services (MeSH): non-medical support services, such as food preparation and bathing, given by trained personnel to disabled, sick, or convalescent individuals in their home.

**References**

1. Case Management Society of America. What is a Case Manager? Available at: http://www.cmsa.org/PolicyMaker/ResourceKit/AboutCaseManagers/tabid/141/Default.aspx. Accessed October 15, 2015.

2. Van Durme T, Schmitz O, Ces S, et al. A comprehensive grid to evaluate case management's expected effectiveness for community-dwelling frail older people: results from a multiple, embedded case study. BMC Geriatr 15:1-13, 2015.

3. Vannieuwenborg L, Buntinx F, De Lepeleire J. Presenting prevalence and management of psychosocial problems in primary care in Flanders. Arch Public Health 73(1):1-6, 2015.

4. World Health Organisation. Clinical Trials. Available at: http://www.who.int/topics/clinical_trials/en/ Accessed October 15, 2015.

5. Institute of Medicine Committee on Evaluating Clinical Applications of T. The National Academies Collection: Reports funded by National Institutes of Health. In: Field MJ, editor. Telemedicine: A Guide to Assessing Telecommunications in Health Care. Washington (DC): National Academies Press (US) National Academy of Sciences, 1996.

6. World Health Organisation. Physical activity. Available at: http://www.who.int/topics/physical_activity/en/ Accessed October 15, 2015.

7. World Health Organisation. Primary Health Care: Main Terminology. Available at: http://www.euro.who.int/en/health-topics/Health-systems/primary-health-care/main-terminology. Accessed May 11, 2016.
