# Supplementary figures and images for: Interventions for frail community-dwelling older adults have no significant effect on adverse outcomes: a systematic review and meta-analysis
Source: BMC Geriatr. 2018 Oct 20;18:249. doi: 10.1186/s12877-018-0936-7 (PMC6195949; doi:10.1186/s12877-018-0936-7)

**Additional Figure S1: Flow chart**

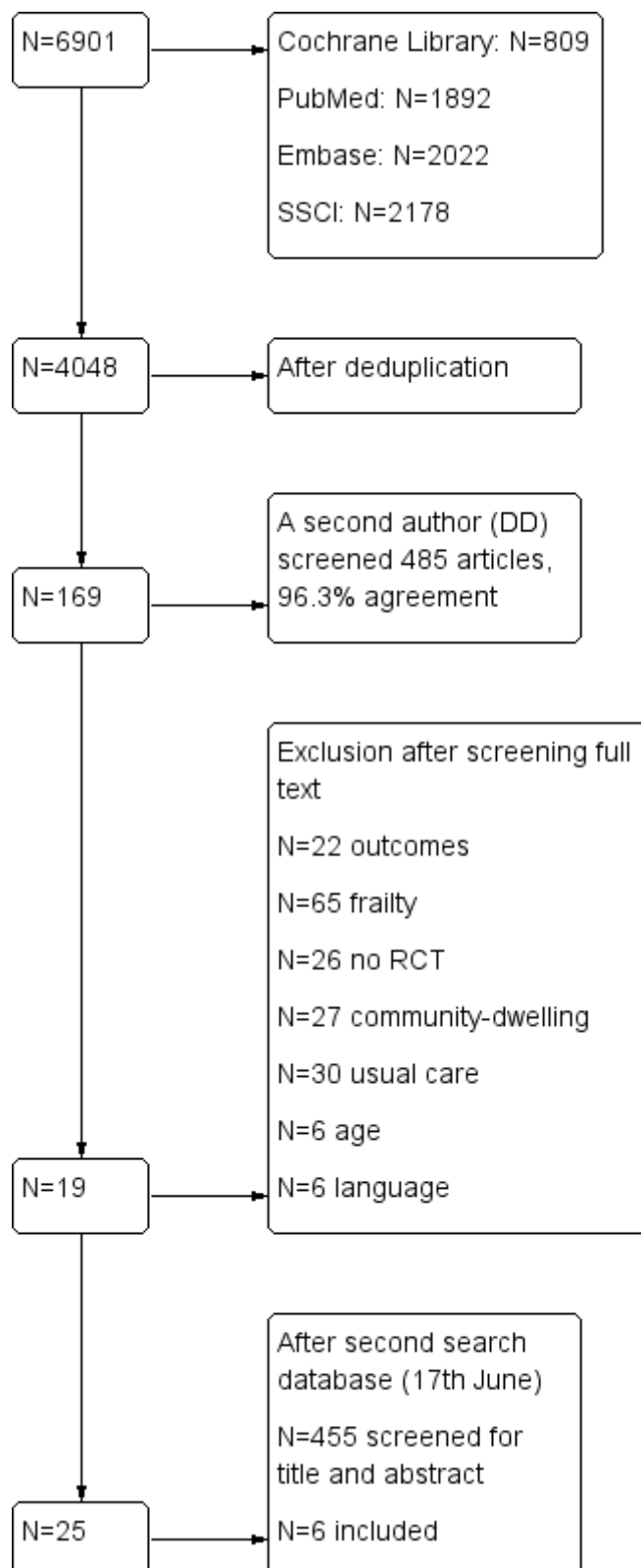

Supplement: Supplementary file 3 — Figure S1. Flow chart (PDF 188 kb) [file 12877_2018_936_MOESM3_ESM.pdf]
