# Supplementary material for: Interventions for frail community-dwelling older adults have no significant effect on adverse outcomes: a systematic review and meta-analysis
Source: BMC Geriatr. 2018 Oct 20;18:249. doi: 10.1186/s12877-018-0936-7 (PMC6195949; doi:10.1186/s12877-018-0936-7)

Additional Figures S2: Forest plot and Funnel plot

Forest plot: intervention case management, outcome mortality

Study or Subgroup	Experimental Events	Experimental Total	Control Events	Control Total	Weight	Odds Ratio M-H, Random, 95% CI
Cameron et al. (2013)	12	116	10	121	6.9%	1.28 [0.53, 3.09]
De Vriendt et al. (2016)	1	86	0	82	0.5%	2.89 [0.12, 72.08]
Favela et al. (2013)	2	45	2	44	1.3%	0.98 [0.13, 7.26]
Favela et al. A (2013)	1	44	2	44	0.9%	0.49 [0.04, 5.59]
Hall et al. (1992)	14	81	18	86	8.9%	0.79 [0.36, 1.71]
Kehusmaa et al. (2010)	12	343	15	365	9.0%	0.85 [0.39, 1.83]
Metzelthin et al (2014)	15	193	10	153	7.8%	1.21 [0.53, 2.76]
Van Hout et al. (2010)	39	331	43	320	25.0%	0.86 [0.54, 1.37]
Van Leeuwen et al. (2015)	20	456	9	227	8.3%	1.11 [0.50, 2.48]
Van Leeuwen et al. A (2015)	34	456	20	238	16.2%	0.88 [0.49, 1.56]
Van Leeuwen et al. B (2015)	43	456	16	226	15.1%	1.37 [0.75, 2.48]
**Total (95% CI)**		**2607**		**1906**	**100.0%**	**0.99 [0.79, 1.25]**
Total events	193		145			

Heterogeneity: Tau² = 0.00; Chi² = 3.50, df = 10 (P = 0.97); I² = 0%

Test for overall effect: Z = 0.07 (P = 0.94)

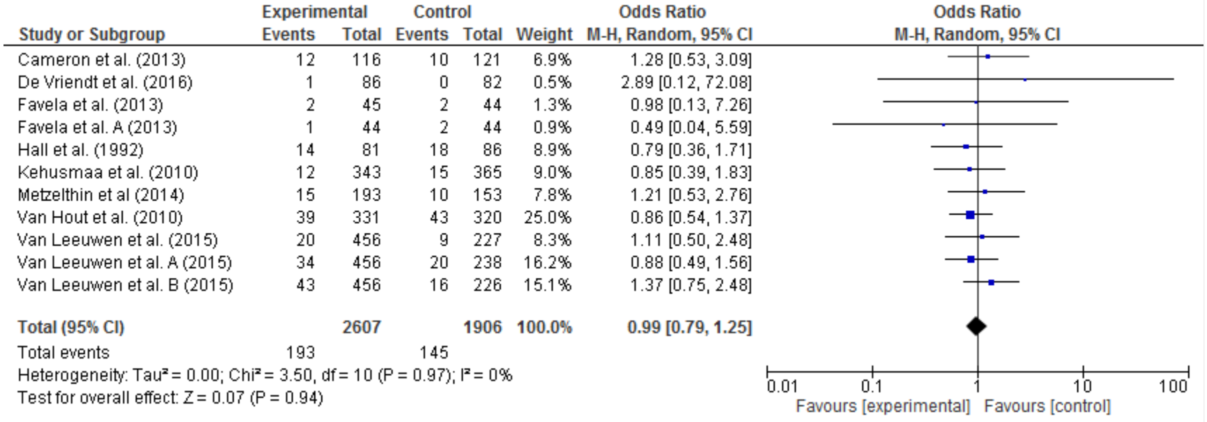

Funnel plot: intervention case management, outcome mortality

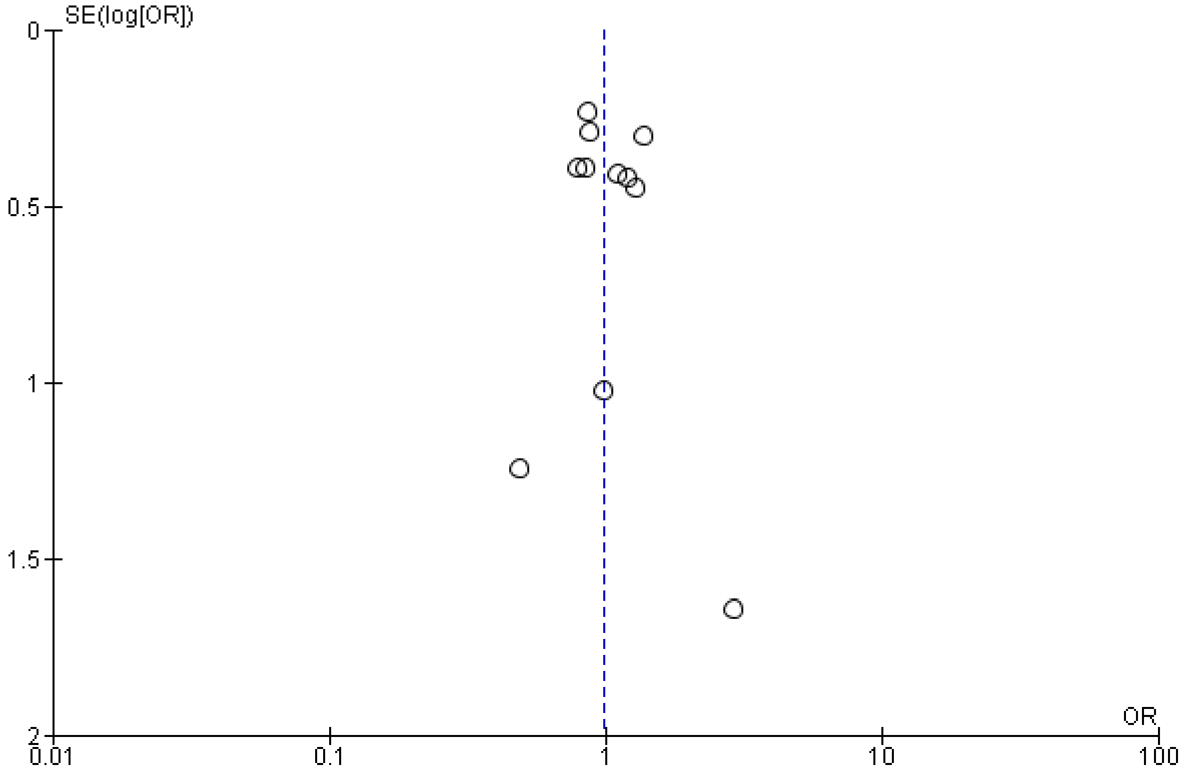

Forest plot: intervention case management, outcome institutionalization

Study or Subgroup	Experimental Events	Experimental Total	Control Events	Control Total	Weight	Odds Ratio M-H, Random, 95% CI
Fairhall et al. (2015)	25	116	30	121	22.7%	0.83 [0.46, 1.53]
Hall et al. (1992)	6	81	17	86	11.2%	0.32 [0.12, 0.87]
Kehusmaa et al. (2010)	41	343	35	365	29.6%	1.28 [0.79, 2.06]
Metzelthin et al. (2015)	5	193	6	153	8.0%	0.65 [0.20, 2.18]
Van Hout et al. (2010)	23	331	20	320	22.0%	1.12 [0.60, 2.08]
Williams et al. (1987)	5	58	4	59	6.4%	1.30 [0.33, 5.09]
**Total (95% CI)**		**1122**		**1104**	**100.0%**	**0.92 [0.63, 1.32]**
Total events	105		112			

Heterogeneity: Tau² = 0.06; Chi² = 7.07, df = 5 (P = 0.22); I² = 29%
Test for overall effect: Z = 0.47 (P = 0.64)

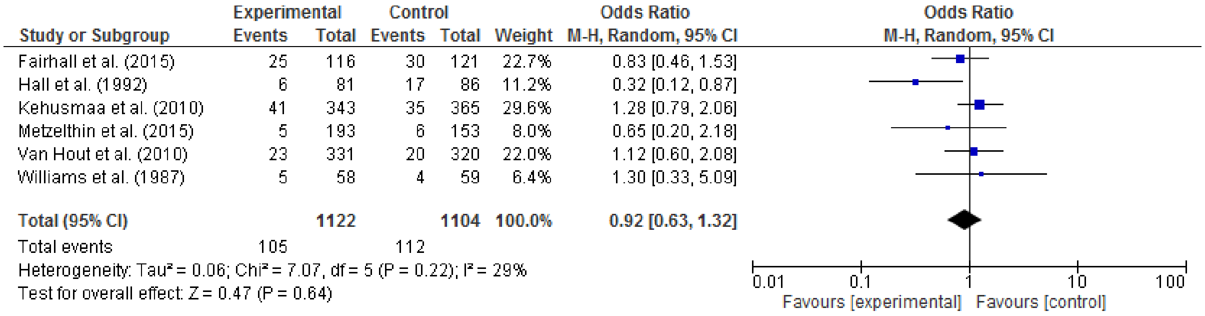

Funnel plot: intervention case management, outcome institutionalization

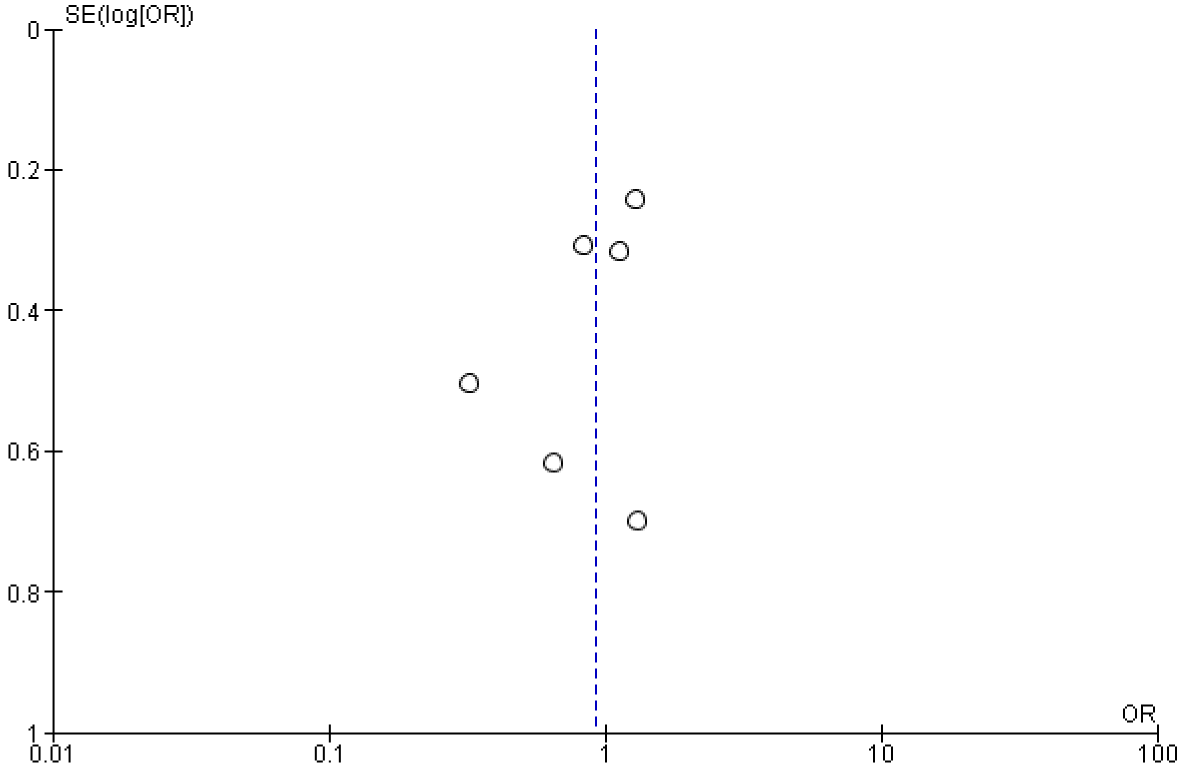

Forest plot: intervention case management, outcome hospitalization

Study or Subgroup	Experimental Events	Experimental Total	Control Events	Control Total	Weight	Odds Ratio M-H, Random, 95% CI
Fairhall et al. (2015)	74	116	66	121	11.9%	1.47 [0.87, 2.47]
Kehusmaa et al. (2010)	184	343	193	365	37.0%	1.03 [0.77, 1.39]
Metzelthin et al. (2015)	39	193	33	153	11.9%	0.92 [0.55, 1.55]
Van Hout et al. (2010)	163	331	141	320	33.9%	1.23 [0.90, 1.68]
Williams et al. (1987)	19	58	18	59	5.3%	1.11 [0.51, 2.42]
**Total (95% CI)**		**1041**		**1018**	**100.0%**	**1.13 [0.95, 1.35]**
Total events	479		451			

Heterogeneity: Tau² = 0.00; Chi² = 2.23, df = 4 (P = 0.69); I² = 0%
Test for overall effect: Z = 1.35 (P = 0.18)

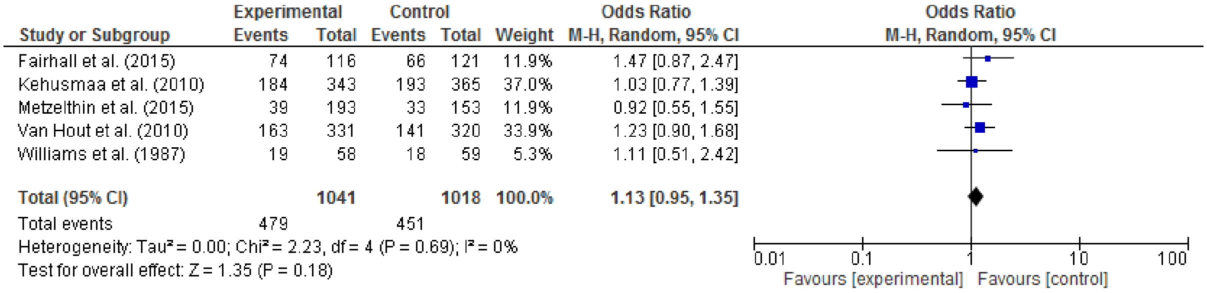

Funnel plot: intervention case management, outcome hospitalization

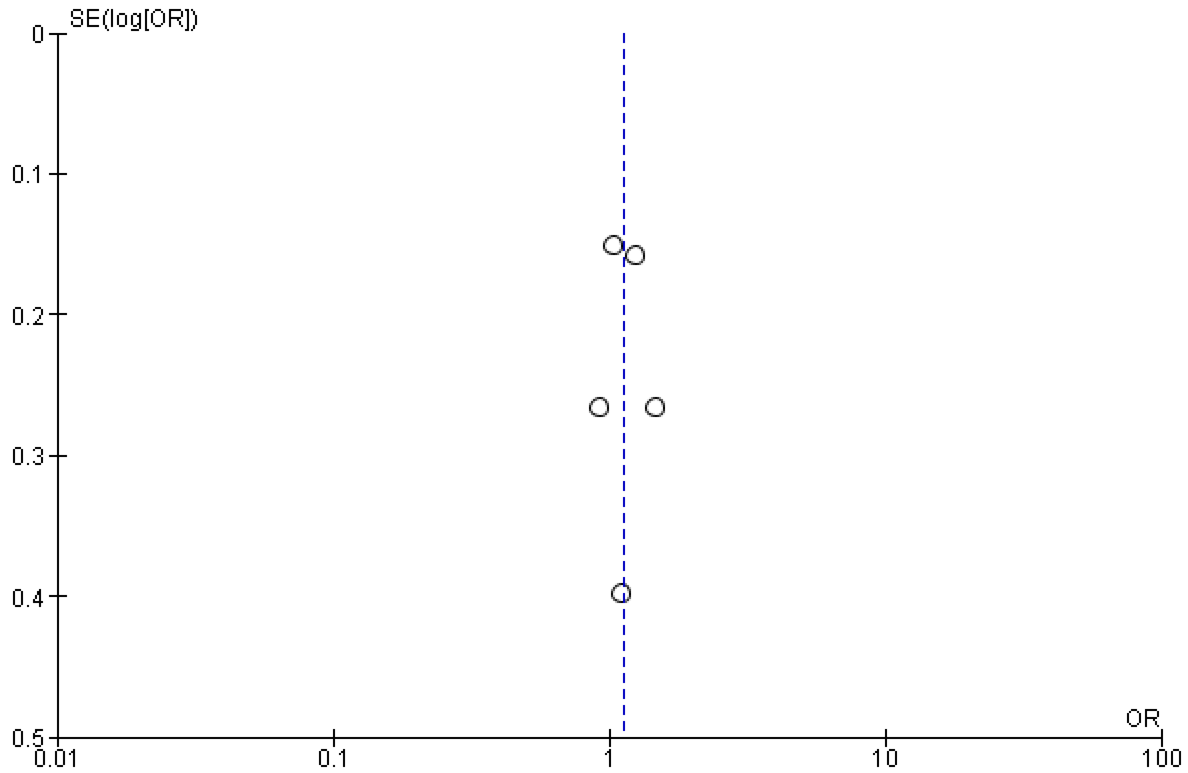

Forest plot: intervention information provision, outcome mortality

Study or Subgroup	Experimental Events	Experimental Total	Control Events	Control Total	Weight	Odds Ratio M-H, Random, 95% CI
Kono et al. (2013)	11	161	20	162	33.3%	0.52 [0.24, 1.13]
Kono et al. (2016)	22	179	17	181	37.9%	1.35 [0.69, 2.64]
Monteserin et al. (2010)	9	151	13	134	28.8%	0.59 [0.24, 1.43]
**Total (95% CI)**		**491**		**477**	**100.0%**	**0.78 [0.41, 1.45]**
Total events	42		50			

Heterogeneity: Tau² = 0.15; Chi² = 4.00, df = 2 (P = 0.14); I² = 50%

Test for overall effect: Z = 0.79 (P = 0.43)

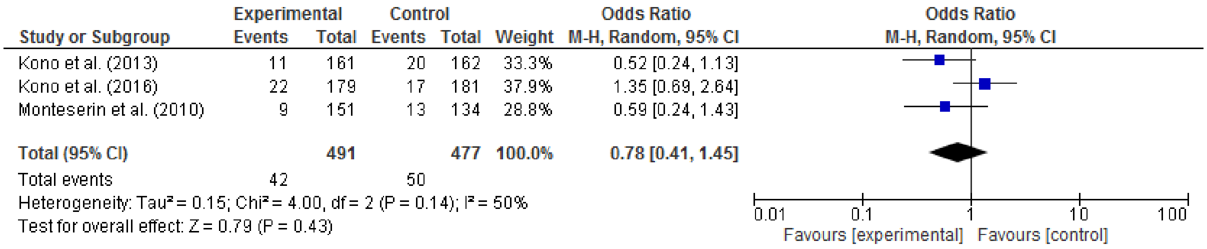

Funnel plot: intervention information provision, outcome mortality

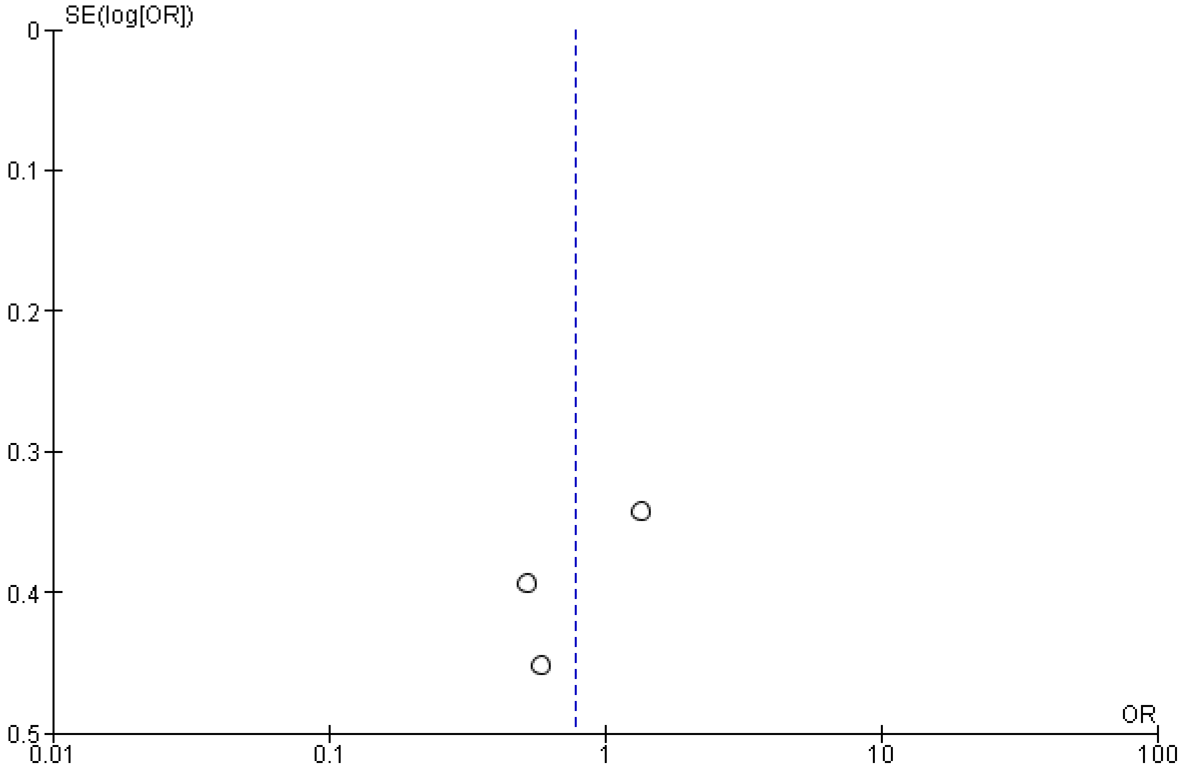

Forest plot: intervention information provision, outcome institutionalization

Study or Subgroup	Experimental Events	Experimental Total	Control Events	Control Total	Weight	Odds Ratio M-H, Random, 95% CI
Kono et al. (2013)	5	161	3	162	36.2%	1.70 [0.40, 7.23]
Kono et al. (2016)	7	179	3	181	40.5%	2.41 [0.61, 9.49]
Monteserin et al. (2010)	2	151	3	134	23.3%	0.59 [0.10, 3.56]
**Total (95% CI)**		**491**		**477**	**100.0%**	**1.53 [0.64, 3.65]**
Total events	14		9			

Heterogeneity: Tau² = 0.00; Chi² = 1.53, df = 2 (P = 0.46); I² = 0%

Test for overall effect: Z = 0.95 (P = 0.34)

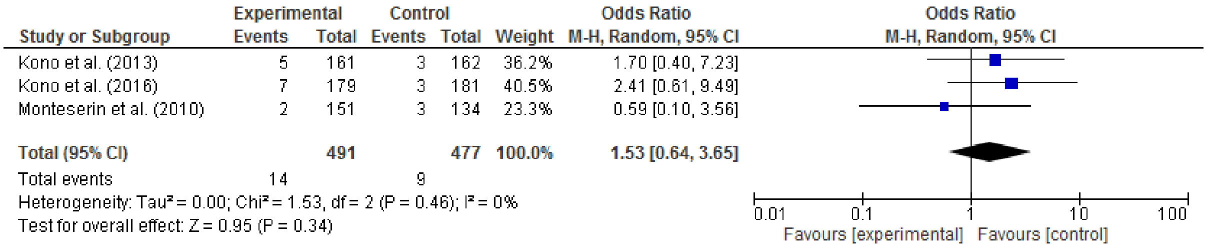

Funnel plot: intervention information provision, outcome institutionalization

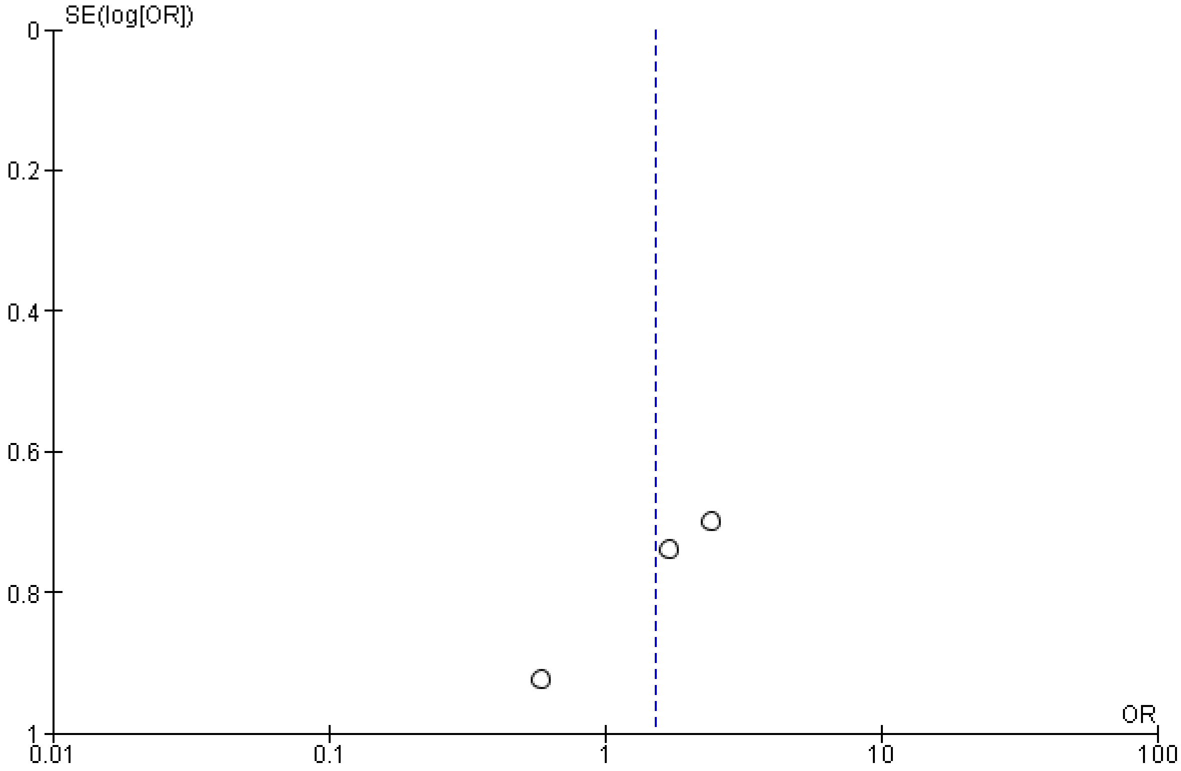

Supplement: Supplementary file 4 — Figure S2. Funnel plot and Forest plot (PDF 257 kb) [file 12877_2018_936_MOESM4_ESM.pdf]
