# Supplementary material for: Interventions for frail community-dwelling older adults have no significant effect on adverse outcomes: a systematic review and meta-analysis
Source: BMC Geriatr. 2018 Oct 20;18:249. doi: 10.1186/s12877-018-0936-7 (PMC6195949; doi:10.1186/s12877-018-0936-7)
Supplement: Supplementary file 5 — Figure S3. Critical appraisal (PDF 187 kb) [file 12877_2018_936_MOESM5_ESM.pdf]

Additional Figure S3: Critical appraisal

	Random sequence generation (selection bias)	Allocation concealment (selection bias)	Blinding of participants and personnel (performance bias)	Blinding of outcome assessment (detection bias)	Incomplete outcome data (attrition bias)	Selective reporting (reporting bias)	Other bias
Cameron et al. (2013)	+	?	?	+	+	+	?
De Vriendt et al. (2016)	+	+	−	+	+	?	?
Dorrestein et al. (2015)	+	+	−	+	+	+	+
Fairhall et al. (2014)	+	?	?	+	+	+	?
Fairhall et al. (2015)	+	?	?	+	+	+	?
Favela et al. (2013)	−	+	−	+	+	?	?
Hall et al. (1992)	+	+	?	+	+	?	?
Kehusmaa et al. (2010)	+	+	?	+	+	?	?
Kim et al. (2015)	+	+	+	+	+	?	−
Kono et al. (2013)	+	+	?	?	?	?	?
Kono et al. (2016)	+	+	−	+	+	+	?
Metzelthin et al. (2014)	−	?	?	+	+	+	?
Metzelthin et al. (2015)	−	?	?	+	+	+	?
Monteserin et al. (2010)	+	+	−	+	?	?	?
Perttila et al. (2016)	+	+	−	−	?	+	?
Upatising et al. (2013)	?	+	−	+	−	?	?
Van Hout et al. (2010)	+	+	−	+	+	?	?
Van Leeuwen et al. (2015)	−	+	−	+	−	+	−
Williams et al. (1987)	+	+	+	+	+	+	?
